# Supplementary material for: A multi-state dynamic process confers mechano-adaptation to a biological nanomachine
Source: Nat Commun. 2022 Sep 10;13:5327. doi: 10.1038/s41467-022-33075-5 (PMC9464220; doi:10.1038/s41467-022-33075-5)
Supplement: Supplementary file 1 — Supplementary Information [file 41467_2022_33075_MOESM1_ESM.pdf]

Supplementary Information for "A multi-state dynamic process confers mechano-adaptation to a biological nanomachine"

Navish Wadhwa[1,2,*,✉], **Alberto Sassi**[3,*], **Howard C. Berg**[4], **and Yuhai Tu**[3,✉]

[1]Department of Physics, Arizona State University, Tempe, AZ 85287

[2]Biodesign Center for Mechanisms of Evolution, Arizona State University, Tempe, AZ 85287

[3]IBM T. J. Watson Research Center, Yorktown Heights, NY 10598

[4]Department of Molecular and Cellular Biology, Harvard University, Cambridge, MA 02138

[*]These authors contributed equally to this work.

✉ Navish.Wadhwa@asu.edu; yuhai@us.ibm.com

Supplementary Note 1.

A plausible model for stator dynamics and its simplified form

Here we present a conceptual model to described the stochastic motion of a bound stator unit to demonstrate a plausible mechanism for the torque-dependence of the off rate. The basic idea is that a bound (tethered) stator unit can become unbound with a unbinding rate $k_{\text{off}}(x)$ that can depend on its displacement x. This dependence of k_{off} on x is reasonable as there are barriers between the bound and unbound states that can depend on location of the stator unit relative to the landing point (the attachment point at $x = 0$). We assume that $k_{\text{off}}(x)$ is maximum at $x = 0$ as the stator unit can unbind through the same pathway as the binding process at $x = 0$ and it decreases as $|x|$ increases, e.g., $k_{\text{off}} = k_0 \exp(-|x|/x_0)$ or $k_{\text{off}} = k_0 \exp\left(-x^2/x_0^2\right)$. This symmetric form of k_{off} for $x \to -x$ is inspired from the CCW and the CW symmetry in stator remodeling[1].

For a tethered stator unit, we can write down the dynamics of its displacement x as a Langevin equation:

$$\frac{dx}{dt} = \xi^{-1}\left(-\frac{dV_0(x)}{dx} - F\right) + \eta, \tag{1}$$

where ξ is the viscosity; η is the thermal noise with $\langle \eta \rangle = 0$ and $\langle \eta(t)\eta(t') \rangle = 2k_BT\xi^{-1}\delta(t-t')$ (k_BT is the thermal energy unit); $V_0(x)$ is the free energy of the tethered stator. If we assume the tethers can be approximated as springs with a spring constant k, we have: $V_0(x) = \frac{1}{2}kx^2$. Of course, the free energy $V_0(x)$ can have more complex landscape with multiple minima separated by energy barriers.

The driving force $F = \Gamma/R$ is due to the counter force from the rotor to the stator unit with Γ the torque and R the rotor

radius. Over a time scale much longer than the time scale of proton translocation through the stator units, Γ may be considered constant. We can then solve Eq. 1 and obtain the steady state distribution of x as:

$$P_s(x) = A\exp[-(V_0(x) + \Gamma x/R)/k_B T], \tag{2}$$

where A is normalization constant. Given the steady state distribution of x given in Eq. 2 and the dependence of the off rate on x given by $k_{\text{off}}(x)$ to determine the observed overall off rate $\tilde{k}_{\text{off}}$:

$$\tilde{k}_{\text{off}}(\tau) = \int P_s(x) k_{\text{off}}(x) dx, \tag{3}$$

which depends on Γ because $P_s(x)$ depends on Γ.

For simplicity, we can coarse grain the continuous x-dependence by two states, **T** and **L**, where the two states are separated by a displacement threshold x_{th}. **L** represents a loose state with $|x| \le x_{\text{th}}$ around $x \simeq 0$ where $k_{\text{off}}(x) \equiv k_{\text{off,l}}$ is large, and **T** represents a tight state at $|x| > x_{\text{th}}$ where $k_{\text{off}}(x) \equiv k_{\text{off,t}}$ is much smaller than $k_{\text{off,l}}$. This simplified model of the bound stator is used in this work.

Supplementary Note 2.

Dwell time statistics in two-state model with only one bound state

If there is only one bound state with a single off rate k_{off} and an on rate k_{on}, the model becomes much simpler. We call this simplified model the two-state model. The survival probability for state-N can be obtained analytically: $S(t) = \exp[-(k_+(N) + k_-(N))t]$ where $k_+(N) = (N_{\text{tot}} - N)k_{\text{on}}$ is the total rate of increasing N by one with k_{on} being the on rate for each binding site, and $k_-(N) = Nk_{\text{off}}$ is the total rate of decreasing N by one with k_{off} being the off rate for each bound stator. From the distribution of survival probability, we can obtain the distribution of dwell times

$$P(\tau) = -\frac{dS}{dt}|_{t=\tau} = (k_- + k_+)\exp[-(k_+ + k_-)\tau], \tag{4}$$

which follows an exponential decay function with a time scale $\tau_{\text{ts}}(N)$ for the two-state model:

$$\tau_{\text{ts}}(N) = (k_+(N) + k_-(N))^{-1}. \tag{5}$$

τ_{ts} depends on N since both $k_+(N) = (N_{\text{tot}} - N)k_{\text{on}}$ and $k_-(N) = Nk_{\text{off}}$ depend on N. However, the range of change in $\tau_{\text{ts}}(N)$ is limited. The ratio between the time scales for any two levels N_1 and $N_2 (< N_1)$ is bounded by:

$$\frac{\tau_{\text{ts}}(N_1)}{\tau_{\text{ts}}(N_2)} = \frac{(N_{\text{tot}} - N_2)k_{\text{on}} + N_2 k_{\text{off}}}{(N_{\text{tot}} - N_1)k_{\text{on}} + N_1 k_{\text{off}}} \le \frac{N_{\text{tot}} - N_2}{N_{\text{tot}} - N_1}, \tag{6}$$

where the equality only exists in the extreme case when $k_{\rm off} = 0$. For example, with $N_{\rm tot} = 12$, the change in time scale in the two-state model between $N_1 = 7$ and $N_2 = 2$ is less than 2: $\frac{\tau_{\rm ts}(7)}{\tau_{\rm ts}(2)} \leq \frac{10}{5} = 2$.

Fig. 3 shows the experimentally determined distribution of dwell times $P_{\rm exp}(\tau|N)$ for different N. The average dwell time $\langle\tau\rangle(N)$ for a given N is then computed from $P_{\rm exp}(\tau|N)$. As shown in **Fig. 3a** in the main text, the experimentally measured timescales $\langle\tau\rangle(N)$ change by over 3-fold as N increases from its low values ($N = 1,2,3$) to high values ($N = 6,7,8$). For example, $\langle\tau\rangle(7)$ = 43.2 s and $\langle\tau\rangle(2)$ = 6.6 s, so $\frac{\langle\tau\rangle(7)}{\langle\tau\rangle(2)} \approx 6.5$, which is much larger than the ratio expected from a two-state model.

For additional comparison, we performed full stochastic simulations of the two-state model for the motor remodeling process starting with 0 bound stator units. We ran the simulation for a duration that matched that of the experiments. The results of these simulations for the dwell time statistics $P(\tau)$ integrated over all values of N for two different values of $k_{\rm off}$ are shown in **Fig.1c** in the main text. Consistent with what we observed in Fig. 3 for individual N, the integrated dwell time distribution $P(\tau)$ from the two-state model also does not agree with the experiments.

The disagreement between the two-state model and experiments can also be seen in the normalized variance magnitude $V = \frac{\int_0^\infty \tau^2 P(\tau) d\tau}{(\int_0^\infty \tau P(\tau) d\tau)^2} - 1$. Using Eqn. 4, one can show that $V = 1$ for a two-state model, whereas $V \approx 2$ was observed in the experiments (**Fig. 3c** in the main text). Furthermore, with only one bound state, the overall off rate $k_-(N) = N k_{\rm off,l}$ becomes independent of time. The distribution functions for the dwell time followed by adding ('+') or removing ('-') of a stator can then be determined analytically for the two-state model: $P_\pm(\tau|N) = k_\pm(N)\exp[-(k_-(N)+k_+(N))\tau]$. In this case, the average dwell times for the '+' (on) and the '-' (off) transitions are equal: $\tau_+(N) = \tau_-(N) = (k_-(N)+k_+(N))^{-1}$, which is again inconsistent with experiments, which show that $\tau_+(N) \neq \tau_-(N)$ (**Fig. 4**).

Taken together, our experimental results rule out the simple two-state model with only one bound state and suggest the existence of multiple bound states with multiple time scales in the remodeling process.

Supplementary Note 3.

Dwell time statistics with two bound states

Here, we determine the dwell time statistics in our proposed model (**Fig. 1b** in the main text) with two bound states (**L** and **T**) by using first-passage time analysis. In this analysis, we neglect the **H**-state that only affects the short time behavior of the system.

Assume at $t = 0$, the motor enters into the state-N with N engaged stator units (by either adding or removing a stator unit). The motor exits the state-N by either having a new stator unit engaged with the rotor (the "on" process) or by having one of the N bound stator units leaving the motor (the "off" process), whichever happens first.

Since the observed transition (either on or off) depends on which transition happens first, the problem is a first passage time problem. The first passage time problem can be treated by computing the survival probability $S(t)$, which is the probability the motor stays in state-N at time $t \geq 0$. Obviously, we have $S(0) = 1$ and $S(\infty) = 0$.

There are two processes (on and off) that terminate the state-N. The on process has a constant (time-independent) rate:

$$k_+ = (N_{\text{tot}} - N)k_{\text{on}}, \tag{7}$$

where N_{tot} is the maximum number of stator units each motor can accommodate, and the on rate k_{on} can depend on the rotational speed ω, which is linearly proportional to the number of stator units ($\omega = \omega_1 N$ in the high load regime with ω_1 the rotational speed per stator unit). Additionally, k_{on} can also depend directly on N due to other mechanisms, such as co-operative interactions.

For $N \geq 1$, the off process has a rate $k_-(t)$ that is more complex as there are multiple stator units each with a different effective "off" rate depending on their arrival time $t_N < t_{N-1} < .. < t_1 \leq 0$ where the newest stator unit arrives at $t_1 \leq 0$. For a given unit-i, the probability of it being still bound with the rotor is its "survival" probability $S_i(t)$. The survived unit-i can be either in the **L** or the **T** state with probability $L_i(t)$ and $T_i(t)$, respectively, and $S_i = T_i + L_i$. $L_i(t)$ and $T_i(t)$ can be determined by solving the following equations:

$$\frac{dL_i}{dt} = -(k_{\text{off,l}} + k_{\text{t}})L_i + k_{\text{l}}T_i, \tag{8}$$

$$\frac{dT_i}{dt} = -k_{\text{l}}T_i + k_{\text{t}}L_i, \tag{9}$$

with the initial conditions $L_i(t_i) = 1$ and $T_i(t_i) = 0$ as we assume the stator is in the L state when it initially binds to the rotor.

Eqs. 8 and 9 can be solved to obtain:

$$L_i(t) = \frac{\sigma_+ - k_{\text{l}}}{\Delta\sigma} e^{-\sigma_+(t-t_i)} + \frac{k_{\text{l}} - \sigma_-}{\Delta\sigma} e^{-\sigma_-(t-t_i)}, \tag{10}$$

$$T_i(t) = \frac{k_{\text{t}}}{\Delta\sigma}(e^{-\sigma_-(t-t_i)} - e^{-\sigma_+(t-t_i)}), \tag{11}$$

$$S_i(t) = T_i(t) + L_i(t) = \frac{\sigma_+ - k_{\text{l}} - k_{\text{t}}}{\Delta\sigma} e^{-\sigma_+(t-t_i)} + \frac{k_{\text{l}} + k_{\text{t}} - \sigma_-}{\Delta\sigma} e^{-\sigma_-(t-t_i)}, \tag{12}$$

where $\sigma_\pm = \frac{1}{2}[(k_{\text{l}} + k_{\text{t}} + k_{\text{off,l}}) \pm \sqrt{(k_{\text{l}} + k_{\text{t}} + k_{\text{off,l}})^2 - 4k_{\text{l}}k_{\text{off,l}}}]$ are the two rate constants (eigenvalues of the rate matrix from Eqs. 8 and 9) with $\sigma_+ > \sigma_- > 0$ and $\Delta\sigma \equiv \sigma_+ - \sigma_- > 0$.

Since a stator unit has a much smaller off rate in the **T** state as compared to the **L** state, i.e., $k_{\text{off,t}} << k_{\text{off,l}}$, by setting $k_{\text{off,t}} = 0$ we can approximate the overall off rate k_- as:

$$k_-(t) = k_{\text{off,l}} \sum_{i=1}^{N} p_i(t) = -\sum_{i=1}^{N} S_i^{-1} \frac{dS_i}{dt}, \tag{13}$$

where $p_i(t) = \frac{L_i}{L_i + T_i}$ is the fractional probability of unit-i to be in the L state at time t.

Given the rates k_+ and k_-, the survival probability satisfies: $S(t+dt) = S(t) \times (1 - (k_+ + k_-)dt)$. By taking the limit

$dt \to 0$, we have:

$$\frac{dS}{dt} = -(k_+ + k_-(t))S, \tag{14}$$

which can be solved with the initial condition $S(0) = 1$:

$$S(t|N) = \exp[-k_+ t] \prod_{i=1}^{N} \frac{S_i(t)}{S_i(0)}, \tag{15}$$

which is the product of the survival probabilities of all the individual bound stator units and the survival probability ($\exp(-k_+ t)$) due to adding a stator unit. Note that in the product we have $\frac{S_i(t)}{S_i(0)}$ to satisfy $S(0) = 1$.

From Eq. 12, the expression for S_i can be written as:

$$S_i(t) = c\exp(-\sigma_+(t - t_i)) + (1 - c)\exp(-\sigma_-(t - t_i)), \tag{16}$$

where $c = \frac{\sigma_+ - k_1 - k_t}{\sigma_+ - \sigma_-}$ is a constant. From the expression of S_i, we can obtain the expression for $\frac{S_i(t)}{S_i(0)}$:

$$S_i(t)/S_i(0) = c_i \exp(-\sigma_+ t) + (1 - c_i)\exp(-\sigma_- t) = e^{-\sigma_- t}[(1 - c_i) + c_i e^{-\Delta\sigma t}], \tag{17}$$

where the coefficient c_i is given by:

$$c_i = \frac{c\exp(\sigma_+ t_i)}{c\exp(\sigma_+ t_i) + (1 - c)\exp(\sigma_- t_i)} = \frac{ce^{\Delta\sigma t_i}}{ce^{\Delta\sigma t_i} + 1 - c}, \tag{18}$$

where $\Delta\sigma \equiv \sigma_+ - \sigma_- > 0$. It is easy to see that $c_i < 1$ and since c can be negative, c_i can also be negative. Given the order of the stator unit arrival times: $t_N < t_{N-1} < ... < t_1 \leq 0$, the absolute value $|c_i|$ decreases with i (in fact, $|c_i|$ decreases exponentially with $|t_i|$).

Finally, the expression for $S(t|N)$ can be written as:

$$S(t|N) = e^{-(k_+ + N\sigma_-)t} \sum_{n=0}^{N} a_n e^{-n\Delta\sigma t}, \tag{19}$$

which contains $(N + 1)$ exponential decay terms each with a different decay rate (or timescale). The coefficients are: $a_0 = \prod_{i=1}^{N}(1 - c_i) > 0$, $a_1 = \sum_{i=1}^{N} c_i \prod_{j\neq i}(1 - c_j)$, $a_2 = \sum_{i=1}^{N} \sum_{j\neq i} c_i c_j \prod_{k\neq i, k\neq j}(1 - c_k)$, etc. Given that c_i can be negative, a_1 can be negative. In principle, a_n can depend on N.

Note that Eq. 19 with $(N + 1)$ timescales is exact. Given the fact that $\Delta\sigma > 0$ and $t_i < 0$, we have $|c_N| < |c_{N-1}| < ... < |c_1| < 1$. For simplicity, we can keep only the larger values of c_i with $i \leq i_m$ and let the other values of c_i to be zero $c_{i>i_m} = 0$. For

example, the simplest model corresponds to $i_m = 1$ and we have:

$$S(t|N) = (1-c_1)e^{-k_a t} + c_1 e^{-k_b t}, \quad N \geq 1 \tag{20}$$

where $k_a = k_+ + N\sigma_-$, $k_b = k_a + \Delta\sigma$, and $S(t) = e^{-k_{+,0}t}$ for $N = 0$. The model then contains three parameters $\sigma_\pm$ and $c_1 \approx c$ if we assume $t_1 \approx 0$.

For the results reported in Figure 3 in the main text, we used $i_m = 3$, i.e., by making the approximation that $c_{i\geq 4} = 0$ and treating c_1, c_2, and c_3 as fitting parameters. This leads to an explicit expression for $S(t|N)$:

$$S(t|N) = \begin{cases} (1-c_1)e^{-k_a t} + c_1 e^{-k_b t}, & N = 1 \\ (1-c_1)(1-c_2)e^{-k_a t} + (c_1 + c_2 - 2c_1 c_2)e^{-k_b t} + c_1 c_2 e^{-k_c t}, & N = 2 \\ (1-c_1)(1-c_2)(1-c_3)e^{-k_a t} + [c_1(1-c_2)(1-c_3) + c_2(1-c_1)(1-c_3) + c_3(1-c_1)(1-c_2)]e^{-k_b t} + & \\ +[c_1 c_2(1-c_3) + c_1 c_3(1-c_2) + c_2 c_3(1-c_1)]e^{-k_c t} + c_1 c_2 c_3 e^{-k_d t}, & N \geq 3 \end{cases} \tag{21}$$

where $k_c = k_a + 2\Delta\sigma$ and $k_d = k_a + 3\Delta\sigma$. The normalization condition $S(0) = 1$ is preserved in the expression for $S(t)$ given above, which has four decay rates (terms) when $N \geq 3$ and there are three variables c_1, c_2, and c_3 that we used to fit the experimental data. Given that the arrival time for the last stator unit $t_1 = 0$ (at least for most cases), we have: $c_1 \approx c$. The value of c reported in the main text is the same as c_1 determined by fitting the model to the experiments.

From the expression of $S(t|N)$ given in Eq. 21, we can obtain the expressions for $k_-(\tau|N) = -\frac{d\ln S}{dt}|_{t=\tau} - k_+$ and the two dwell time distributions $P_+(\tau|N)$ and $P_-(\tau|N)$ for the '+' and '-' transitions when the bound stator number is N:

$$P_+(\tau|N) = k_+(N)S(\tau|N), \quad P_-(\tau|N) = k_-(\tau|N)S(\tau|N)\,. \tag{22}$$

The overall dwell-time distribution $P(\tau|N) = P_+(\tau|N) + P_-(\tau|N)$. These dwell time distributions can be used to derive expressions for $\langle\tau\rangle$, f_+, and V for each N:

$$\langle\tau\rangle(N) = \int_0^\infty \tau P(\tau|N)d\tau\,, \tag{23}$$

$$f_+(N) \equiv \frac{\int_0^\infty P_+(\tau|N)dt}{\int_0^\infty P(\tau|N)d\tau}\,, \tag{24}$$

$$V(N) \equiv \frac{\int_0^\infty \tau^2 P(\tau|N)d\tau}{(\int_0^\infty \tau P(\tau|N)d\tau)^2} - 1\,, \tag{25}$$

which depend on $k_+(N)$, σ_-, $\Delta\sigma$, c_1, c_2 and c_3.

Supplementary Note 4.

Possible role of cell-to-cell variability

Here we consider the possible effects of cell-to-cell variability on the statistics of dwell times and in particular on the variability in dwell times for a given stator unit number N. If there is a large enough variability in rates, especially k_+ due to cell-to-cell variation in the number of freely diffusing stator units, then even within a 2-state model such cell-to-cell variability could potentially account for the large observed values of $V \equiv \frac{\langle\tau^2\rangle}{\langle\tau\rangle^2} - 1$, where τ is the dwell time. Such cell-to-cell variability would increase V. However, as we show below, the large value of V (≈ 2) observed in our experiments can not be explained by cell-to-cell variability because that would require an unrealistically high level of variability in the kinetic rates in a highly correlated fashion.

In a 2-state model, the statistics of dwell time are determined by the total rate $k = k_+ + k_-$, which is the sum of the on-rate (k_+) and the off-rate (k_-). For a given k, τ follows an exponential distribution: $P(\tau|k) = k\exp(-k\tau)$ with its first and second moments given by $\langle\tau\rangle = k^{-1}$ and $\langle\tau^2\rangle = 2k^{-2}$, respectively. Now, let us assume that the cell-to-cell variation in k can be described by a distribution $q(k)$ for k. We can then express the normalized variance V as:

$$V \equiv \frac{\langle\tau^2\rangle}{\langle\tau\rangle^2} - 1 = \frac{\int 2k^{-2}q(k)dk}{(\int k^{-1}q(k)dk)^2} - 1. \tag{26}$$

For simplicity, let us assume that k has a broad uniform distribution between k_1 and k_2, i.e., $q(k) = (k_2 - k_1)^{-1}$ when $k_1 \leq k \leq k_2$ and 0 otherwise. This distribution is characterized by a maximum fold change $f = k_2/k_1 \geq 1$. From Equation 26, one can show that V depends on the fold change f as:

$$V = \frac{2(f-1)^2}{f(\ln f)^2} - 1, \tag{27}$$

which has a rather weak dependence on f (see Fig. 5 above). When $f \to 1$, i.e., there is no variation in k, $V \to 1$. V increases with f, but slowly. For V to reach the observed value of ≈ 2, f has to be as large as 10. For a more reasonable value of $f = 4$, we have $V = 1.34$, which is far below the observed value of V.

Another important issue is that since $k = k_+ + k_-$, a large fold change in k requires both k_+ and k_- to have the same (or similar) fold change in a correlated way. If the variations in k_+ and k_- are uncorrelated, which we believe is the case here, the variation in k measured by fold change will be dominated by the rate that has the higher average value. At higher value of N, k_- is comparable to and even larger than k_+, in which case the variation in k could be dominated by variation in k_-. However, within the 2-state model, there is no obvious reason for k_- to have a large variability. For example, if we assume that k_- has a relatively small variation and the averages of k_+ and k_- are the same ($\bar{k}$), then even a highly variable distribution for k_+, e.g., a bimodal distribution $P(k_+) = [\delta(k_+) + \delta(k_+ - 2\bar{k})]/2$ with a maximum fold change for k_+, $\frac{k_{+,max}}{k_{+,min}} \to \infty$ would only lead to a modest fold change in k: $f \approx 3$, which is far below what is needed to achieve $V \approx 2$ within the 2-state model.

Supplementary Note 5.

The model parameters are obtained with an optimization algorithm

Given that we can derive analytical expressions for the relevant quantities using the model equations, we implemented a gradient descent algorithm to find the rate parameters leading to the best agreement between the theory and the experimental results. More precisely, we tried to find the values of $k_+(N)$, c_i $(i = 1,2,3)$, σ_+ and σ_-, as defined in the previous sections, that would lead to values of $\langle\tau\rangle$, f_+ and $\langle V\rangle_N = \left\langle \sigma_\tau^2/\langle\tau\rangle^2 \right\rangle_N$ where $\langle ...\rangle_N$ indicates an average over N, that are closest to what can be directly obtained from experiments. To do this, we define a vector parameter p, whose components are the parameters that we are trying to fit, and a loss function L defined as

$$L(p) = \sum_{N=0}^{9} \left[\alpha_1 (\langle\tau\rangle^{exp} - \langle\tau\rangle^{mod}(p,N))^2 + \alpha_2 (f_+^{exp} - f_+^{mod}(p,N))^2 \right] + \alpha_3 \left(\frac{1}{9} \sum_{N=0}^{9} \sigma_\tau^2/\langle\tau\rangle^2 - V^{mod} \right)^2 , \tag{28}$$

where α_1, α_2 and α_3 are three constant weights. At every step the parameter vector is updated according to

$$p \to p - \eta \cdot \nabla_p L , \tag{29}$$

where η is a constant learning rate. Here, with *exp* and *mod* we indicate the mean values of each quantity as measured from the experimental data or as obtained from the model equations respectively.

The choices of the parameters α_i $(i = 1,2,3)$ depend on the accuracy that we want to have in the fitting of the three experimental variables. In our case we chose $\alpha_1 = 1/\sigma_\tau^2$, where $\sigma_\tau^2 = \langle\tau^2\rangle - \langle\tau\rangle^2$, $\alpha_2 = 1$ and $\alpha_3 = 10$.

The fit of our model to the experimental data shown in **Fig. 3** in the main text led to the parameters $c_1 = 0.30 \pm 0.11$, $c_2 = 0.12 \pm 0.14$, $c_3 = 0.06 \pm 0.16$, $\sigma_+ = 0.19 \pm 0.1$, $\sigma_- \leq 0.0005$. The errors correspond to the fluctuation in the value of the parameter that would lead to a 50% increase in the loss function.

The parameters may also be obtained or further refined by using the maximal likelihood estimation (MLE), which fits the full distribution that includes in principle all the higher order moments of the distribution. Unless the model is inconsistent with the data, the "adjustment" of these parameters is typically small and they may not lead to a better fit of the physically meaningful properties of the system such as $\langle\tau\rangle$ or f_+.

We performed MLE for our problem, and indeed it does not change the results significantly. In particular, we performed the optimization again using a MLE loss function of the following form:

$$L_{MLE}(p,x) = \sum_N \sum_i D[P^{exp}(x, i\,dt, N), P^{th}(p, i\,dt, N)] + \sum_N (f_+^{exp}(x) - f_+^{th}(p))^2 , \tag{30}$$

where x are the experimental data, p the fitting parameter, P^{exp} and P^{th} the dwell time distributions for a given number of stator units, D is a measure of the distance between the two functions and dt the bin size we chose to evaluate the distributions. We used

the mean squared difference here for D. Other distance measures such as the log likelihood function (or the Kullback–Leibler divergence) lead to poor fitting of the distribution and $\langle\tau\rangle$ because these distance measures tend to overemphasize the outliers in the dwell time distribution at large τ where the noise in the measured distribution is high.

We used the gradient descent method to minimize L_{MLE} given above and the parameters obtained are only slightly different from those we reported in the article, namely $c_1 = 0.39$ (0.3 ± 0.1 in the article), $c_2 = 0.14$ (0.12 ± 0.14 in the article), $c_3 = 0.012$ (0.06 ± 0.16 in the article), $\sigma_+ = 0.09$ (0.19 ± 0.1 in the article). Although the predicted values for τ, f_+, and V from these adjusted parameters agree with experiments within the experimental error bars, the agreement is not improved in comparison with those shown in Fig. 3 in the main text with parameters determined by minimizing the moment-based loss function of Eq. 28. Since the adjustment in parameters was small and the fitting to $\langle\tau\rangle$ and f_+ is not improved, we did not use the MLE approach in our study.

Supplementary Figures

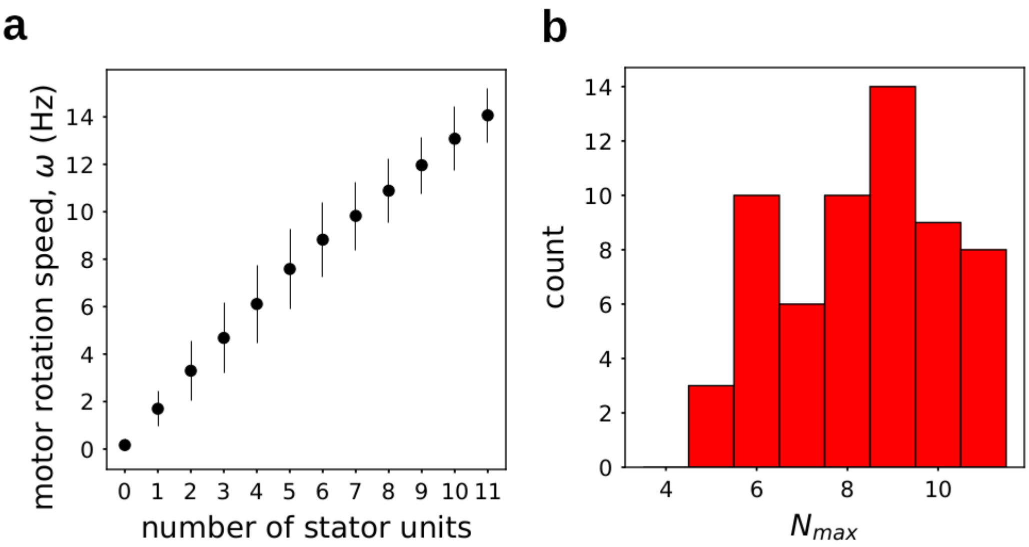

Supplementary Figure 1. a. Average rotation speed vs number of stator units. Error bars represent standard deviation. **b.** Histogram of the largest number of bound stator units reached in an experiment. We have $N_{\mathrm{max}} \leq N_{\mathrm{tot}}$, where $N_{\mathrm{tot}} = 11$ is the maximal number of stator units that can be accommodated in a flagellar motor.

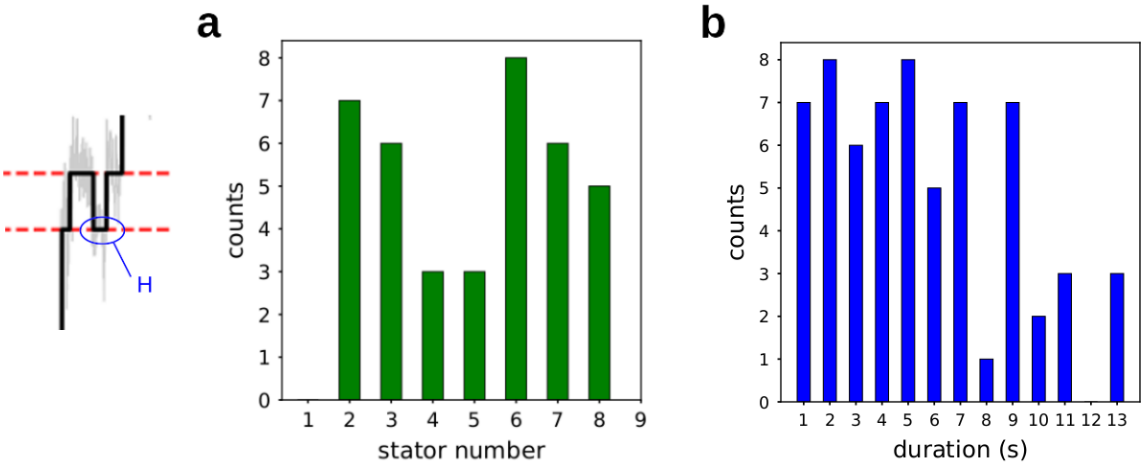

Supplementary Figure 2. Occurrence of short "wells" for different stator numbers due to transition to the short-lived unbound state. A short well is defined as an unbinding event followed by a binding event within $\Delta\tau < 8$ s. **a.** Counts of short wells observed at different stator units numbers. **b.** Total counts of wells observed for different values of the threshold duration ($\delta\tau$) of the well.

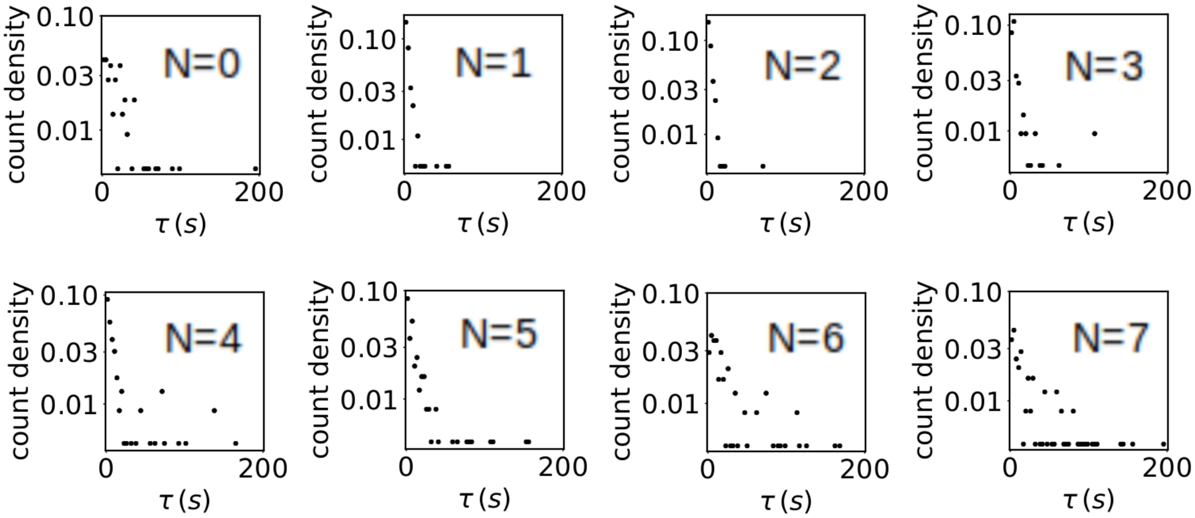

Supplementary Figure 3. Semi-log plot of the dwell time distributions for different values of $N = 0, 1, 2, ..., 7$ (the number of bound stator units).

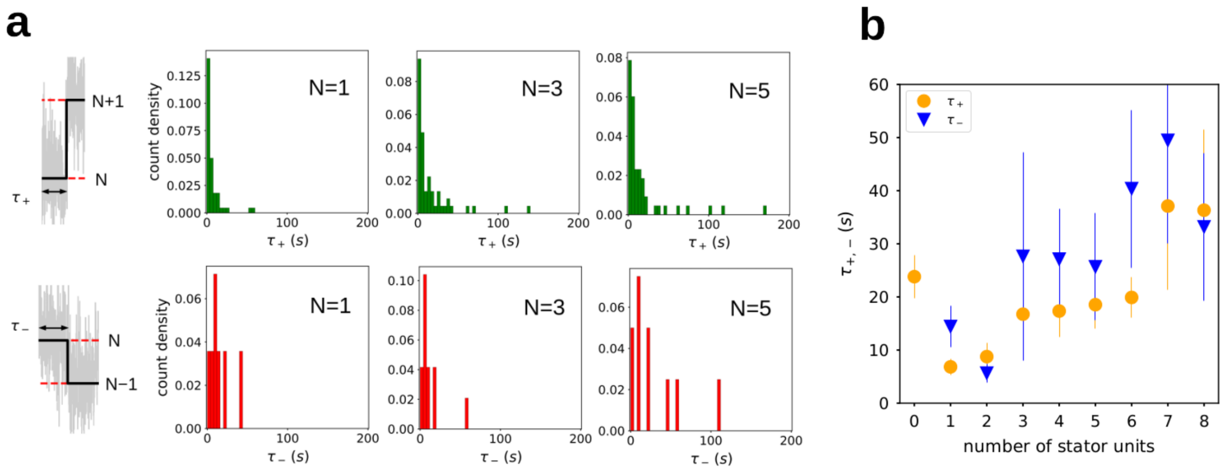

Supplementary Figure 4. a. Histograms of dwell times ending with binding (above) and unbinding (below) of a new stator unit, for three different values of N. **b.** Conditional dwell times τ_+ (yellow circle) and τ_- (blue triangle) from the electrorotation experiments vs the number of stator units bound to the rotor. Data presented are the means and the error bars represent standard deviation. The sample size for determining τ_+ for 0, 1, 2,... 8 stator units is 74, 51, 54, 60, 54, 67, 52, 54, 37, respectively. The sample size for determining τ_- for 1, 2,... 8 stator units is 12, 21, 10, 23, 14, 28, 29, 37, respectively.

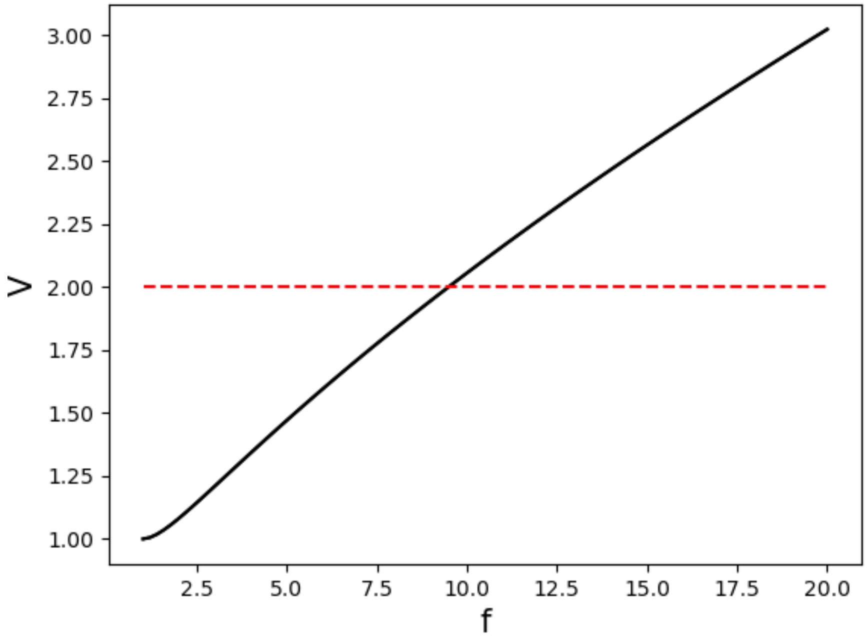

Supplementary Figure 5. The dependence of the normalized variance V on the maximum fold change f of the total transition rate ($k = k_+ + k_-$) due to motor-motor variability (see Supplementary Text). A large value of $f \approx 10$ is required in order to have $V = 2$ (the dashed line).

Supplementary References

1. Wadhwa, N., Tu, Y. & Berg, H. C. Mechanosensitive remodeling of the bacterial flagellar motor is independent of direction of rotation. *Proc. Natl. Acad. Sci.* **118** (2021).
