# Supplementary material for: A multi-state dynamic process confers mechano-adaptation to a biological nanomachine
Source: Nat Commun. 2022 Sep 10;13:5327. doi: 10.1038/s41467-022-33075-5 (PMC9464220; doi:10.1038/s41467-022-33075-5)
Supplement: Supplementary file 3 — Reporting Summary [file 41467_2022_33075_MOESM3_ESM.pdf]

nature research

Corresponding author(s): Navish Wadhwa; Yuhai Tu

Last updated by author(s): Aug 17, 2022

Reporting Summary

Nature Research wishes to improve the reproducibility of the work that we publish. This form provides structure for consistency and transparency in reporting. For further information on Nature Research policies, see our Editorial Policies and the Editorial Policy Checklist.

Statistics

For all statistical analyses, confirm that the following items are present in the figure legend, table legend, main text, or Methods section.

n/a	Confirmed	
☐	☒	The exact sample size (n) for each experimental group/condition, given as a discrete number and unit of measurement
☐	☒	A statement on whether measurements were taken from distinct samples or whether the same sample was measured repeatedly
☒	☐	The statistical test(s) used AND whether they are one- or two-sided<br>*Only common tests should be described solely by name; describe more complex techniques in the Methods section.*
☒	☐	A description of all covariates tested
☐	☒	A description of any assumptions or corrections, such as tests of normality and adjustment for multiple comparisons
☐	☒	A full description of the statistical parameters including central tendency (e.g. means) or other basic estimates (e.g. regression coefficient) AND variation (e.g. standard deviation) or associated estimates of uncertainty (e.g. confidence intervals)
☒	☐	For null hypothesis testing, the test statistic (e.g. F, t, r) with confidence intervals, effect sizes, degrees of freedom and P value noted<br>*Give P values as exact values whenever suitable.*
☒	☐	For Bayesian analysis, information on the choice of priors and Markov chain Monte Carlo settings
☒	☐	For hierarchical and complex designs, identification of the appropriate level for tests and full reporting of outcomes
☒	☐	Estimates of effect sizes (e.g. Cohen's d, Pearson's r), indicating how they were calculated

Our web collection on statistics for biologists contains articles on many of the points above.

Software and code

Policy information about availability of computer code

Data collection	Data collection was automated using a custom-written program in LabView 2017 SP-1.
Data analysis	Custom-written codes in Python 3.6.4 were used for data analysis.

For manuscripts utilizing custom algorithms or software that are central to the research but not yet described in published literature, software must be made available to editors and reviewers. We strongly encourage code deposition in a community repository (e.g. GitHub). See the Nature Research guidelines for submitting code & software for further information.

Data

Policy information about availability of data

All manuscripts must include a data availability statement. This statement should provide the following information, where applicable:

- Accession codes, unique identifiers, or web links for publicly available datasets
- A list of figures that have associated raw data
- A description of any restrictions on data availability

The raw data used in all analyses presented here are available from the GitHub repository https://github.com/navishwadhwa/multi-state-remodeling. Source data for Figs. 1c, 3 and 4 are provided with this paper.

Field-specific reporting

Please select the one below that is the best fit for your research. If you are not sure, read the appropriate sections before making your selection.

☒ Life sciences ☐ Behavioural & social sciences ☐ Ecological, evolutionary & environmental sciences

For a reference copy of the document with all sections, see nature.com/documents/nr-reporting-summary-flat.pdf

Life sciences study design

All studies must disclose on these points even when the disclosure is negative.

Sample size	Sample size was not chosen a priori, but a total of 58 independent experiments were analyzed in which there was relatively little cell-to-cell variation in the overall outcome of the experiment (increased number of stator units to saturation). These data yielded hundreds of measurements of dwell time, which was the key quantity used for downstream analysis and modeling.
Data exclusions	Data exclusion was largely performed after calculating rotation speed traces. Those traces (approximately 10) which were too noisy for reliable step-fitting were excluded from further analysis.
Replication	Since each rotation speed trace was an independent experimental replicate, no additional replication was performed.
Randomization	Cells used for experiments were randomly selected from a culture of millions of cells. Each flow cell contained hundreds of single cells of which only a few were selected for experiments on any given day.
Blinding	The key results are derived from a single set of raw data so blinding was not possible or relevant.

Reporting for specific materials, systems and methods

We require information from authors about some types of materials, experimental systems and methods used in many studies. Here, indicate whether each material, system or method listed is relevant to your study. If you are not sure if a list item applies to your research, read the appropriate section before selecting a response.

Materials & experimental systems

n/a	Involved in the study	
☒	☐	Antibodies
☒	☐	Eukaryotic cell lines
☒	☐	Palaeontology and archaeology
☒	☐	Animals and other organisms
☒	☐	Human research participants
☒	☐	Clinical data
☒	☐	Dual use research of concern

Methods

n/a	Involved in the study	
☒	☐	ChIP-seq
☒	☐	Flow cytometry
☒	☐	MRI-based neuroimaging
